# New bladder cancer non-invasive surveillance method based on voltammetric electronic tongue measurement of urine

**DOI:** 10.1016/j.isci.2022.104829

**Published:** 2022-08-04

**Authors:** Javier Monreal-Trigo, Miguel Alcañiz, M. Carmen Martínez-Bisbal, Alba Loras, Lluís Pascual, José Luis Ruiz-Cerdá, Alberto Ferrer, Ramón Martínez-Máñez

**Affiliations:** 1 Instituto Interuniversitario de Investigación de Reconocimiento Molecular y Desarrollo Tecnológico (IDM), Universitat Politècnica de València - Universitat de València, Camino de Vera, s/n, 46022 Valencia, Spain; 2 CIBER de Bioingeniería, Biomateriales y Nanomedicina, Instituto de Salud Carlos III, Madrid, Spain; 3 Departamento de Electrónica, Universitat Politècnica de València, Camino de Vera, s/n, 46022 Valencia, Spain; 4 Departamento de Química Física, Universitat de València, C/ Doctor Moliner, 50, 46100 Burjassot, Valencia, Spain; 5 Unidad Mixta de Investigación en Nanomedicina y Sensores, Universitat Politècnica de València – Instituto de Investigación Sanitaria La Fe, 46026 Valencia, Spain; 6 Unidad Mixta UPV-CIPF de Investigación en Mecanismos de Enfermedades y Nanomedicina, Universitat Politècnica de València, Centro de Investigación Príncipe Felipe, 46012 Valencia, Spain; 7 Departamento de Medicina, Universitat Jaume I, 12071 Castellón, Spain; 8 Departamento de Cirugía, Hospital Universitario y Politécnico La Fe, 46026 Valencia, Spain; 9 Servicio de Urología, Hospital Universitario y Politécnico La Fe, 46026 Valencia, Spain; 10 Departamento de Estadística e Investigación Operativa Aplicadas y Calidad, Universitat Politècnica de València, Camino de Vera, s/n, 46022 Valencia, Spain; 11 Grupo de Ingeniería Estadística Multivariante, Universitat Politècnica de València, Camino de Vera, s/n, 46022 Valencia, Spain; 12 Departamento de Química, Universitat Politècnica de València, Camino de Vera, s/n, 46022 Valencia, Spain

**Keywords:** Computer-aided diagnosis method, Cancer, Devices

## Abstract

Bladder cancer (BC) is the sixth leading cause of death by cancer. Depending on the invasiveness of tumors, patients with BC will undergo surgery and surveillance lifelong, owing the high rate of recurrence and progression. In this context, the development of strategies to support non-invasive BC diagnosis is focusing attention. Voltammetric electronic tongue (VET) has been demonstrated to be of use in the analysis of biofluids. Here, we present the implementation of a VET to study 207 urines to discriminate BC and non-BC for diagnosis and surveillance to detect recurrences. Special attention has been paid to the experimental setup to improve reproducibility in the measurements. PLSDA analysis together with variable selection provided a model with high sensitivity, specificity, and area under the ROC curve AUC (0.844, 0.882, and 0.917, respectively). These results pave the way for the development of non-invasive low-cost and easy-to-use strategies to support BC diagnosis and follow-up.

## Highlights

• Bladder cancer (BC) and control urines were studied by voltammetric electronic tongue
• A PLSDA model was obtained with high sensitivity, specificity, and accuracy (84/88/86)
• 103/122 BC urines and 7⅝5 control urines were predicted correctly
• The electronic tongue has the potential for non-invasive BC diagnostics and follow-up

## Graphical abstract

**Figure:**
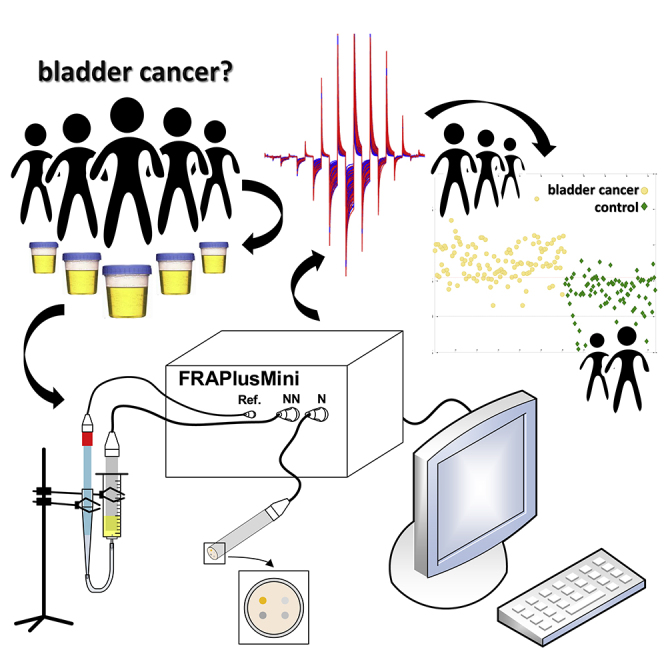

## Introduction

Bladder cancer (BC) is the sixth leading cause of death by cancer, with serious social and economic consequences (Leal et al., 2016). At the time of diagnosis, the tumor stage is classified in two categories: muscle-invasive bladder cancer (MIBC) and non-muscle-invasive bladder cancer (NMIBC). MIBC comprises tumors with a stage ≥ T2, which account for ∼20% of the diagnoses. On the other hand, NMBIC encompasses carcinoma *in situ* (CIS), which are mucosa-contained flat-shaped high-grade lesions, Ta tumors, which are papillary tumors enclosed in the mucosa, and T1 tumors, which present *lamina propria* invasion. CIS is the least common NMIBC at diagnosis, with a 10%, followed by T2 (20%) and Ta (70%) (van Rhijn et al., 2009).

BC prognosis is radically different according to BC muscle-invasiveness: MIBC 5-year survival is <50% and its management involves radical cystectomy and cycles of radiotherapy, immunotherapy, and chemotherapy as adjuvants. NMBIC 5-year survival is ∼90%, involving transurethral resection (TUR) with posterior intravesical instillation with bacillus Calmette-Guérin or mitomycin. NMIBC recurrence rate is one of the highest of all cancers: 50%–70% recurrence in the first two years after diagnosis and 10%–15% progress to invasive within 5 years (Burger et al., 2013; Park et al., 2014). This explains the NMIBC management requiring lifetime follow-up by routinely cystoscopy and urinary cytology, high sensibility and specificity methods not without limitations: they are invasive, operator-dependent, involve high costs, and present low accuracy in the detection of low-grade tumors and CIS, which are considered tumors at high risk of progression (Babjuk et al., 2017; Rosser et al., 2013; Su et al., 2019; Tang and Chang, 2015).

The development of non-invasive methods for early diagnosis and recurrences-detection in the post-diagnosis and post-surgery surveillance period would simplify the clinical procedures as well as decrease the related economic costs. Urine, due to its contact with the bladder, is a logical approach to find BC biomarkers (Yang et al., 2017). Several urinary diagnostic tests based on cytogenetics or proteins for BC diagnosis and/or follow-up have received the FDA approval (Lodewijk et al., 2018; Mbeutcha et al., 2016). In particular, liquid biopsy, genomics, proteomics, glycomics, and metabolomics for the discovery of biomarkers have gained importance in the recent years (Bardelli and Pantel, 2017; Bujak et al., 2015; Di Meo et al., 2017; Feng et al., 2013). Focusing on metabolomics, the quantification of metabolites, the downstream product of gene expression with importance in transcription, translation, and other upstream molecular processes (Burgio et al., 2010; Ladurner, 2006), offers dynamic biochemical information that can be linked to the phenotype of the disease, supporting the use of metabolites and metabolic profiles as a source of biomarkers of disease, which has merged as widely informative to tumor biology (Emwas et al., 2013; Kaushik and DeBerardinis, 2018; Patel and Ahmed, 2015). NMR and mass spectrometry (MS) studies focused on the BC biomarkers identification using urine and blood have obtained good sensitivities and specificities (≈80%–100%) comparing samples from patients with BC tumor versus samples from healthy controls or patients free of cancer (Cheng et al., 2015; Hong et al., 2015; Issaq et al., 2008; Loras et al., 2018, 2019b; Shao et al., 2017; Zhang et al., 2018).

NMIBC follow-up would be the most benefited from the development of a non-invasive reliable alternative to cystoscopy and urinary cytology because of its life-long, several-times-a-year surveillance tests. Pathway analyses show taurine, alanine, aspartate, glutamate, and phenylalanine perturbed metabolism associated with NMIBC with >80% sensibility and specificity (Loras et al., 2019a). Even though NMR spectroscopy and MS experiments require highly qualified personnel, the results are operator-dependent and imply high costs and limited accessibility because of the complexity of the measurement equipment.

A different approach can take advantage of the metabolic pathways information obtained in the previous studies and overcome the mentioned limitations of NMR and MS-based diagnosis and monitoring. Electronic tongues (e-tongues) integrate a system of non-specific sensors whose response can be analyzed with a pattern recognition algorithm or multivariate analysis (Winquist, 2008), presenting remarkable capabilities for classification and quantification of physicochemical parameters. Application examples range from quality assessment in food (Cetó et al., 2016; Peris and Escuder-Gilabert, 2016; Tan and Xu, 2020) water (Cetó et al., 2016), environmental studies (Cetó and Valle, 2022) to pharmaceutics (Wasilewski et al., 2019), and biomedicine (Belugina et al., 2021; Braz et al., 2022; Lvova et al., 2009; Pascual et al., 2016; Solovieva et al., 2019) among others.

**Figure 1:**
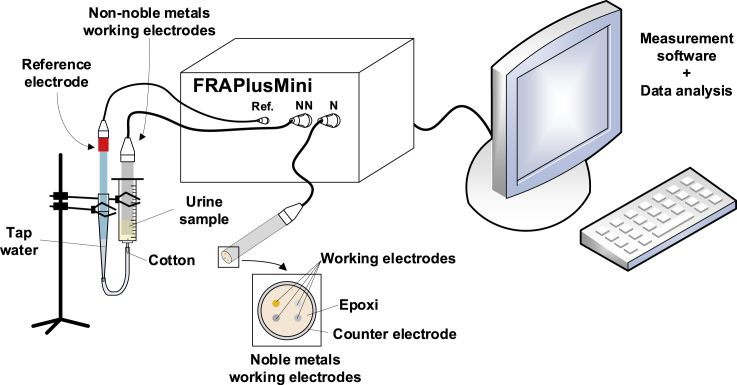
Voltammetric electronic tongue setup The figure includes the different components for the measuring system including the potentiostat (FRAPlusMini), the sensors with the working electrodes (Ir, Rh, Pt, and Au in the noble metals working electrodes array, connected by “N” to the potentiostat, and Ag, Cu, Co, and Ni in the non-noble metals working electrodes array, connected by “NN” to the potentiostat), the counter electrode, the reference electrode (connected by “Ref.” to the potentiostat), the system with the software for measurement and data analysis, and the sample. The figure also shows the detail on the setup for the urine sample and the electrodes by using a syringe, connected to the reference electrode, and the detail in the electrode array entailing the counter electrode and four working electrodes, whose disposition is shown in the figure for the noble metals array.

In particular, the most widespread and to which most of the examples cited correspond, mainly because of their simplicity, robustness, versatility, and high sensitivity to redox reactions among other characteristics, are the voltammetric electronic tongues (VET) (Winquist et al., 1997). Their principle of operation consists of the application of a set of potential pulses to different metallic electrodes and the sampling of the resulting current. Therefore, a VET is conformed mainly by four elements: the electrode system, the pulse applied, the electronic system, and the data analysis algorithm, as shown in Figure 1. Mainly, each generated current can be explained according to the contribution of three components: the diffusion of charges by electrostatic attraction/repulsion, the linear components by the physical properties of the electrical system (i.e. its equivalent capacitance, inductance, and resistivity), and the oxidation-reduction reactions that may take place at the interface between the electrode and the dissolution. Electronic tongues have been applied in the medical field for the determination of urea and creatinine in urine (Gutiérrez et al., 2007; Saidi et al., 2018), amino acids (Faura et al., 2016), biological objects (Kolisnichenko et al., 2014), of endotoxins, and biological contaminants (Heras et al., 2010), for the detection of dengue disease (Welearegay et al., 2020), bacterial infections (Al Ramahi et al., 2019), and dysfunctions in the urinary system (Lvova et al., 2009), among other applications. In the latter study, Lvova et al. used a potentiometric tongue for the diagnosis of BC in a set of 27 urines from patients with BC and healthy controls, all of them male, and observed that a non-supervised analysis (a PCA model) provided a clear separation between urines from BC and healthy controls (Lvova et al., 2009). More recently, Belugina et al. have reported good results (71%, 58%, and 72% of sensitivity, specificity, and accuracy, respectively) to discriminate between BC and healthy controls in a study of 87 urine samples with a potentiometric e-tongue (Belugina et al., 2021), with improved results when age ranged between 50 and 88 years (80%, 75%, and 76% of sensitivity, specificity, and accuracy, respectively) and using a urine volume of 10 mL. In the context of urological cancer, the diagnosis of prostate cancer (PCa) has also been an issue of interest for e-tongues. A sensitivity and specificity >90% for the discrimination of PCa and healthy controls was obtained by Solovieva et al. with a potentiometric e-tongue in a set of 89 urines (Solovieva et al., 2019). VET has shown also a high sensitivity (91%) and specificity (73%) for the diagnosis of PCa in a set of 114 urines of patients with PCa and healthy controls, and are also promising for the application of this tool in the BC field (Pascual et al., 2016). Nevertheless, the context in the study of PCa and BC is different. The interest on BC diagnosis is not only to identify the disease at the moment of diagnosis but also to identify the disease along the follow-up to detect recurrences which are frequent after surgery as mentioned before. Besides, the reduction of sample volume needed for the study becomes also relevant to accomplish the requirements of availability of sample for different analyses, and also for storage in biobanks.

Based on the above, we report herein a novel implementation of a VET to measure low volume of urine sample for BC diagnosis and surveillance in a set of 207 samples, trying to overcome the limitations of NMR and MS: the dependence on the skilled operator and the high cost (in equipment installation, maintenance, and operation). BC urines included samples from male and female patients at diagnosis before surgery and in the follow-up after surgery to detect recurrences. Voltammograms for eight working electrodes were obtained measuring in 1.5 mL of urine in a specially designed setup to provide reproducible geometry of the working, counter, and reference electrodes. Measurements were performed without any dilution. PLSDA (Barker and Rayens, 2003) and variable selection by using variable importance projection (VIP) scores were used to calculate a model to classify BC from control urines, with high sensitivity, specificity, and area under the ROC curve AUC (0.844, 0.882, and 0.917, respectively). These results show the potential of VET as an economic, fast, and easy-to-use tool to support BC identification in the diagnosis and in the follow-up.

## Results and discussion

Urine samples (207 samples) were included in the BC (85), or non-BC (122) sets according to the cystoscopy and urinary cytology analyses. After thawing, samples were measured at room temperature. VET measurements were performed by using a specially designed setup to study small sample volumes (1.5 mL) and to assess a reproducible position for the working electrodes and the counter electrode (electrodes array) with respect to the reference electrode. This last aspect has been shown relevant to ensure reproducibility, avoiding variability due to the position of the electrodes during the measurements (Bataller et al., 2015). Moreover, despite urine being a biofluid easy to obtain, the number of complementary tests performed in each sample is increasing, so, usually the total volume is split in aliquots and the final volume available is reduced. By using the syringe to connect the reference electrode outside the place of measurement, only a small volume of sample was needed to provide surface enough in contact with working and counter electrodes, and ensured a regular, equidistant, and reproducible position of the reference electrode. A scheme of the setup is shown in the Figure 1.

**Figure 2:**
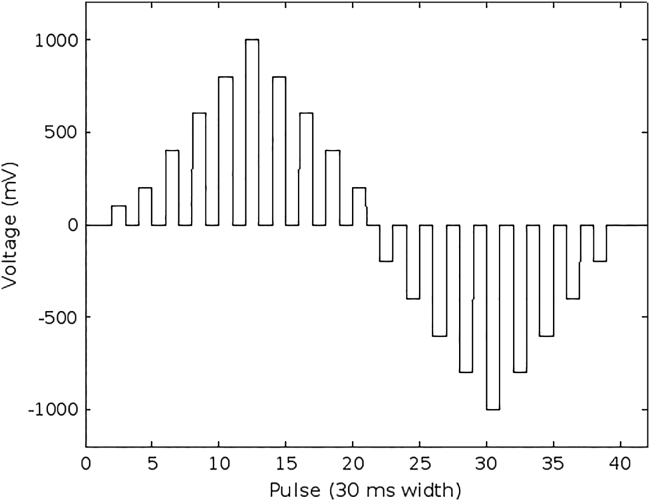
Pulse train applied to one electrode The pulse train was applied to the urine samples using each of the eight metal electrodes, with a total of 42 pulses. Each pulse was set for 30 ms, and the length of the complete train of pulses for an electrode was 1260 ms. The figure shows the voltage in mV applied for each pulse of 30 ms width.

The measurements were performed using a large amplitude pulse voltammetry (LAPV) waveform (Winquist, 2008) that consisted on 42 pulses of 30 ms ranging from −1000 to 1000 mV. The pulses were sequentially applied through the eight working electrodes (Ir, Rh, Pt, Au, Ag, Cu, Co, and Ni) and voltammograms for the 207 samples were obtained. Figure 2 shows the LAPV waveform applied.

**Figure 3:**
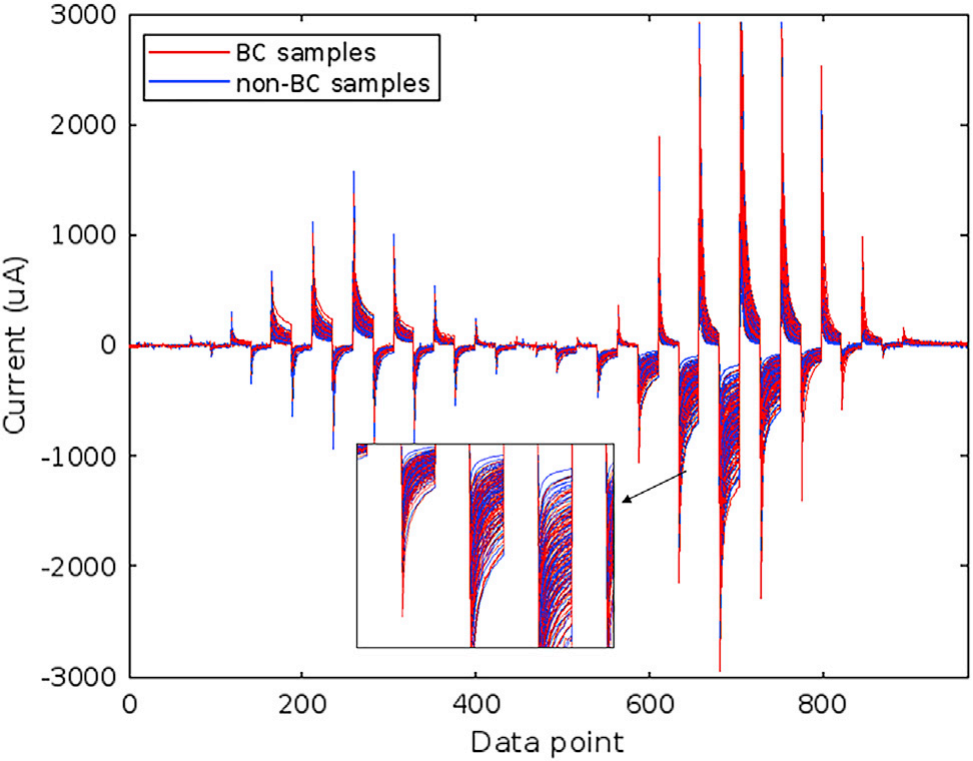
Voltammetry measurements for Pt electrode The figure shows the voltammetry measurements of Pt electrode for all samples. Discrete number of current data points are represented in X axis and current intensity in μA is represented in Y axis. The urines are colored according to the class as BC urines in red and non-BC urines in blue. A region has been magnified to observe the distribution of signals in the pulses.

**Figure 4:**
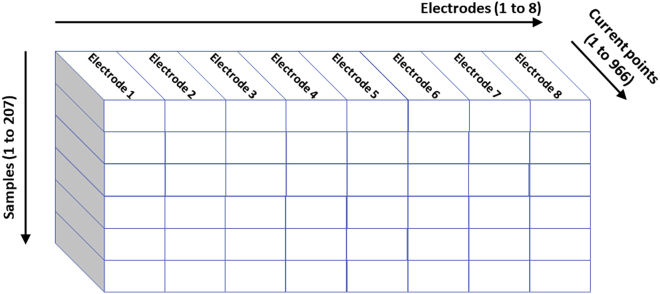
Three-dimensional structured dataset The data are structured in 966 current points by each of the eight electrodes, and for each of the 207 urine samples.

For each pulse, 23 current data points were recorded. Five iterations were programmed for each sample and electrode. A total of 7728 currents were recorded in each iteration. Figure 3 shows the voltammograms for the Pt working electrode for BC and non-BC urines shown in different color and the Figure 4 shows the three-dimensional structured dataset.

**Figure 5:**
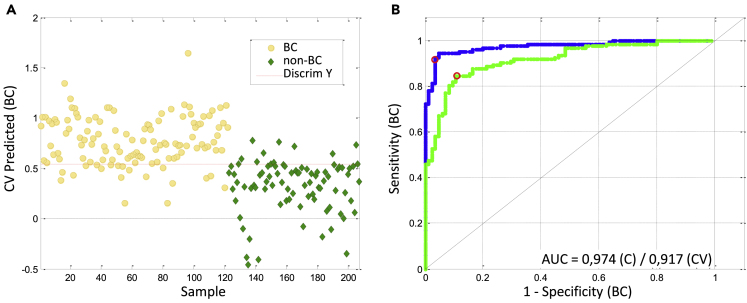
PLSDA prediction and AUC for the model calculated with VIP >1.9 (A) PLSDA prediction scores are represented for BC urines in yellow and non-BC urines in green for the model with VIP>1.9 and 300 variables. (B) Estimated (“C” blue) and Cross-Validated (“CV” green) ROC curves (sensitivity for BC discrimination vs. 1-Specificity) are also displayed together with the AUC values for both “C” and “CV”.

The data from the fifth iteration were selected for analysis and underwent multivariate statistics. Before statistical analysis, data were normalized trying to minimize the influence of different urine concentration. A first PLSDA model was calculated with the normalized and autoscaled dataset that included 7728 variables. Once generated this model (Calibration, C), cross validation (CV) was performed to select the most appropriate number of latent variables. The model thus obtained (raw model) presented a limited performance for BC classification with a sensitivity of 0.566, a specificity of 0.635, and an AUC of 0.638 (Table 1). Then, the raw model was improved with variable selection, including only those variables with VIP score >1.9 (300 variables). The threshold for VIP score was selected according to the highest value of AUC obtained for CV. This improved model achieved a sensitivity of 0.844, a specificity of 0.882, and an AUC of 0.917 for the CV. The quality parameters for both PLSDA models (raw model with 7728 variables and VIP >1.9 model with 300 variables) are shown in the Table 1. To assess the statistical significance of the models, permutation tests were performed, showing that the model calculated from VIP >1.9 was statistically discriminant at the 95% confidence level (Table 1). The classification threshold was set to 0.542, with 1 corresponding to BC urines and 0 corresponding to non-BC urines. PLSDA and ROC curve for this model are shown in Figure 5.

**Table 1:** Quality parameters for PLSDA models

	variables	LV	Calibration^a^			Cross validation^a^			Permutation test^b^		
			Sensitivity	Specificity	AUC	Sensitivity	Specificity	AUC	W	S	R
Raw	7728	3	0.689	0.871	0.812	0.566	0.635	0.638	0.148	0.197	0.330
VIP >1.9	300	3	0.918	0.965	0.974	0.844	0.882	0.917	0.009	0.040	0.010

a Sensitivity, Specificity, and AUC are expressed relative to the highest value of one.

b W, Wilcoxon test; S, Sign test; R, Rand t-test p values. Value less than 0.05 indicates the model is statistically discriminant at the 95% confidence level and are shown in bold.

**Figure 6:**
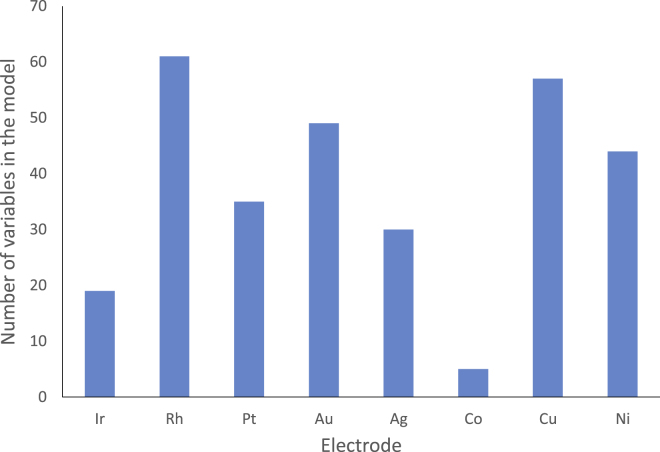
Distribution of data in the model VIP >1.9 according to the electrodes The number of variables belonging to each of the eight metallic working electrode for the final model with 300 variables after VIP >1.9 selection is presented in bars.

A revision of the variables (currents) included in the model VIP >1.9 was performed to determine the participation of each working electrode in the model. It turned out that all the metallic electrodes participated in the model, with a number of variables ranging from 5 to 61 data until complete the 300 variables included in the model. Figure 6 shows the distribution of variables in the final model according to the working electrode. The Co electrode participated only with five variables and the Rh electrode provided the highest participation in the model with 61 variables. 54.7% of the data in the model came from noble working electrodes, and a 45.3% came from non-noble working electrodes. The Ni electrode which is newly implemented here compared to previously published data (Pascual et al., 2016) participated with 44 variables for BC discrimination.

The complete set of features of the model could be calculated according to the confusion matrix data, shown in the Table 2. The model predicted correctly 103 BC urines and 75 non-BC urines, and predicted 19 false negatives and 10 false positives. According to these data, the model specifications of utility for clinical application are shown in Table 3 expressed in percentage, and with the confidence interval at 95%.

**Table 2:** Confusion matrix for VIP >1.9 model

	BC	non-BC
Predicted as BC	103	10
Predicted as non-BC	19	75

**Table 3:** Model specifications

Parameter	Value (%)	IC^a^ 95%	
Sensitivity	84.4	77.0	89.8
Specificity	88.2	79.7	93.5
Predictive positive value	91.2	84.5	95.1
Predictive negative value	79.8	70.6	86.7
False positives	11.8	6.5	20.3
False negatives	15.6	10.2	23.0
Accuracy	86.0	80.6	90.1

a IC, Confidence interval.

High sensitivity, specificity, and accuracy were obtained with this model. Nevertheless, in order to evaluate the results obtained by VET, it is necessary to compare it with the gold standard in sensitivity and specificity (∼>90%): the urinary cytology and cystoscopy (Kamat et al., 2016), which present the limitations stated in the introduction and are therefore used in the follow-up and in case of suspicion. As an alternative non-invasive diagnostic and follow-up method, urine analysis by NMR spectroscopy or MS has obtained promising results (sensitivity and specificity ∼>80%) (Loras et al., 2018, 2019a). It should be noted that in the clinical routine one of the most widespread diagnostic methods for screening is the analysis of metabolites in urine, which has high specificity (90%) but low sensitivity (55%). Besides sensitivity and specificity, other features are of importance in the comparison between techniques for diagnosis and surveillance, as the invasiveness, the costs, or the requirements for specialized staff. The comparison between all these methods is shown in Table 4.

**Table 4:** Advantages and disadvantages of different methods to detect BC

Parameter	UC & C^a^	NMR spectroscopy^b^	MS^c^	UMA^a^	VET
Sensitivity	0.87	0.86	0.82–0.70	0.55	0.84
Specificity	0.99	0.81	0.91–0.75	0.90	0.88
Non-invasive	No	Yes	Yes	Yes	Yes
Inexpensive	No	No	No	No	Yes
Easy to perform	No	No	No	No	Yes
Portable equipment to perform measurements and data analyses *in situ*	No	No	No	No	Yes

a UC & C, urinary cytology and cystoscopy, and UMA, urine metabolite analysis, data from Kamat et al. (2016).

b NMR spectroscopy data from Loras et al. (2019a).

c MS data from Loras et al. (2018).

Comparing with other techniques, as shown in Table 4, VET offers sensitivity and specificity in the order of those required for BC screening (i.e. ∼> 80%). The advantages of easy collection and preparation of the sample, without causing disturbances to the patient and the measurements can be performed by non-specialized staff, the low cost of the equipment and consumables, that can be implemented to perform measurements and data analyses *in situ* make of VET an appealing technique to translate to the clinical routine.

Regarding to the previously published data on e-tongues for urological cancer discrimination, the results here shown, obtained from a set of 207 urine samples from male and female population, and in patients previous to the surgery and in the follow-up for recurrence, bring the application of VET closer to the requirements in the clinical scenario of BC. The sensitivity, specificity, and accuracy have been improved compared to previously published data on e-tongue for BC discrimination (Belugina et al., 2021; Lvova et al., 2009). Moreover, the use of only 1.5 mL is an advantage compared to the 10 mL used in other e-tongues. Also deserves to be mentioned the fact that the set up here presented enables the study of this reduced volume without any dilution, which improves the results of this technique. Finally, the Ni working electrode has been here demonstrated to be important in the discriminant model, which was not used previously in the study of PCa by VET (Pascual et al., 2016).

## Limitations of the study

The potential of the VET for this application has been demonstrated by this work, but for its clinical application it is necessary to pay attention to the metabolites identified in the last studies of the group, redesigning the train of pulses to be applied and, if necessary, the electrodes that compose it. As seen in the study, the participation of the working electrodes is not the same, and it might be considered to simplify the number of working electrodes for future improvements. Moreover, in order to transfer this strategy as a support to diagnosis in the clinical environment, it will also be necessary to use disposable electrodes with high reproducibility to avoid the necessary conditioning processes in the electronic tongue used in this study.

## STAR★Methods

### Key resources table

**Table:** 

REAGENT or RESOURCE	SOURCE	IDENTIFIER
**Software and algorithms**		

Solo	https://eigenvector.com	Version 8.9

### Resource availability

#### Lead contact

Further information and requests for resources and/or reagents should be directed to and will be fulfilled by Prof. M.Carmen Martínez-Bisbal with email: carmen.martinez-bisbal@uv.es.

#### Materials availability

This study did not generate new unique reagents.

### Experimental model and subject details

#### Urine samples

218 urine samples were obtained in the urological service in Hospital Universitario y Politécnico La Fe (Valencia, Spain) from 154 patients under the legal consent procedures (Ethics Committee for Biomedical Research of the Instituto de Investigación Sanitaria Hospital Universitario y Politécnico La Fe, approval number 2012/0186). 11 samples were discarded as having an uncertain anatomopathological diagnosis. A total of 207 urine samples were studied using VET, coming from BC patients before or after surgery. Urines from patients before surgery were included in cancer group (BC). Regarding to the urines collected after surgery, patients were included in follow up procedures to detect recurrences, thus urine samples were taken at monthly intervals after surgery. Cystoscopy and urinary cytology were used to detect BC recurrence. Finally, considering the urines before and after the surgery, in the 207 samples, 85 (41%) were urines from patients free of cancer and 122 urines (59%) were urines from patients with BC. According to the stage of the first tumour, BC cancers were classified as Tx (unidentified, 2 sample), Tis (in-situ carcinoma, 1 samples), Ta (pre-invasive papillary carcinoma, 45 samples), T1 (lamina propria invasive tumour, 62 sample), and T2 (moderate muscle-invasive tumour, 11 samples).

The urine samples, after being collected, were frozen and stored at -80°C until analysed. The procedure prior to analysis consisted of thawing at room temperature (21°C), centrifugation at 2500 rpm for 5 minutes (to remove solids and other insoluble materials) and aliquoting. The samples were managed (processed, conserved and supplied) by Biobanco La Fe, a biobank authorized and certified according to the requirements of Real Decreto 1716/2011 (ref. PT13/0010/0026), and the standard ISO 9001:2008. An aliquot of 1.5 mL was reserved for VET measurements.

### Method details

#### Electronic voltammetric tongue

In this study an array of eight metallic working electrodes (Ir, Rh, Pt, Au, Ag, Co, Cu and Ni) with a purity of 99.99% and a 2-mm diameter (GoodFellow) was used to perform VET study. The metallic electrodes were encapsulated in two stainless steel cylinders, one encapsulating noble metals (Ir, Rh, Pt and Au) and another encapsulating semi noble metals (Ag, Co, Cu and Ni) respectively. In turn, the stainless cylinders were used as the counter electrode and together with noble (Ir, Rh, Pt and Au) or non-noble (Ag, Co, Cu and Ni) working electrodes constituted the noble or non-noble electrodes array. A saturated calomel electrode was used as the reference electrode. The Figure 1 shows the equipment and the experiment setup scheme. The electronic equipment employed allows the programming of pulse voltammetry with up to 50 pulses with an amplitude ranging from -2 to +2 V and a pulsewidth from 1 to 800 ms. This equipment is commanded through USB with its software application and was developed in Instituto Interuniversitario de Investigación de Reconocimento Molecular y Desarrollo Tecnológico – IDM, Universitat Politècnica de València (UPV, Valencia, Spain) (Garcia-Breijo et al., 2013).

Before each measurement, the working electrodes were conditioned. An adequate conditioning process of the electrodes is of importance to provide reproducible data. The process here applied has been different for noble and non-noble metals. Noble metal electrodes were electropolished following previously published procedures (Bataller et al., 2016; Sobrino-Gregorio et al., 2018). Briefly, the noble metals array underwent a sequence of cathodic and anodic pulse in acidic or basic solution to remove the organic material. The process for noble metals has been previously optimised for automatisation and details have already been published (Sobrino-Gregorio et al., 2018) and patented (Bataller et al., 2016). Non-noble metal electrodes were mechanically polished. Non-noble metals were manually conditioned by using sequentially a sanding disc and a fine finishing disc of 1500 and 6000 grain respectively (3M Trizact Clearcoat Sanding Disc 1500 and Hookit Trizact 6000) for 1 minute each of them. After mechanical polishing, the non-noble metals array was rinsed with distilled water.

In order to allow a reproducible contact between the urine sample and all the electrodes (working electrode, counter electrode and reference electrode), and considering the small volume of sample available, the measurements were performed using a 20 mL syringe (Louer-Lok syringe, BD) with centred whole for the needle. The plunger and the needle were discarded, and the barrel was used to contain the sample and the working electrodes. A small quantity of cotton covered the centred tip at the end of the barrel, connected throughout the salt bridge to the reference electrode, thus avoiding the matter exchange between the sample and the salt bridge, and enabling the electric current transference. The reference electrode was inserted into a 5 mL pipette tip and connected through a 5 mm silicone tube to the tip of the barrel, filled with cotton. The space between the reference electrode and the cotton was filled with tap water as saline bridge. For each measurement, the sample (1.5 mL of urine) was introduced in the syringe barrel, and the noble electrodes array were introduced in the barrel, submerging always the same surface of the working electrodes and counter electrode. After the noble electrodes measurement, they were removed from the syringe, and then non-noble electrodes array was introduced in the syringe and the measurements were performed with them. A complete scheme of the system setup is shown in Figure 1.

A Large Amplitude Pulse Voltammetry (LAPV) waveform (Winquist, 2008) was applied to all eight working electrodes, composed of 42 pulses of 30 ms ranging from −1000 to 1000 mV. The LAPV waveform was applied five times to each urine sample. Each iteration gathered information of 23 current data of 42 pulses in each of the 8 electrodes, thus 7728 current data by each sample. The fifth iteration was considered for data analysis.

### Quantification and statistical analysis

Current response to each voltage impulse, in each electrode, according to the variations of the metabolic pathways of multiple substances may require multivariate analysis for the design of a robust classification algorithm offering reliable diagnosis. The whole dataset collected by the VET consist of 207 samples with 966 current points per each of the eight working electrodes. As the application of voltage and current measurement to the electrodes takes place simultaneously, the operation of the VET can be described in real time as eight univariate functions, one for each of the electrodes, with 966 current points each. In this way, the data can be structured three-dimensionally: in samples, current points, and electrodes, as shown in Figure 4.

Multivariate analysis was performed by the PLSDA method (Barker and Rayens, 2003) using the software PLS Toolbox Solo 8.9 (Eigenvector Research, Inc.) for chemometric analysis. Before the statistical analysis, data were preprocessed. Normalisation was performed to avoid the influence of different urine concentration. Data from each electrode was divided by the data from the points in the sixth pulse (400 mV) of the corresponding electrode. After normalisation, data were and autoscaled (every of the 7728 current points in sample was set to unit variance). Once preprocessed, the data underwent PLSDA analysis to determine a model to discriminate between BC and non-BC urines. Cross validation (CV) was performed by using Venetian Blinds (maximum number of latent variables was 20, 10 data splits, and 1 sample per blind) to select the optimum number of latent variables (LV) for the model. The model was subsequently simplified and improved by selecting the variables according to the Variable Prediction Importance score (VIP). The models with variables with VIP scores threshold from 1 to 2 were calculated. To determine the optimal threshold for VIP score, the AUC for CV was calculated, being the highest AUC provided by the best the model. AUC is considered a robust statistic in medical diagnostics (Hajian-Tilaki, 2013). Sensitivity, specificity and AUC were used in the evaluation of both models calculated.

To assess the robustness of the calculated models, permutation tests (Lindgren et al., 1996) were performed by using 200 iterations. Wilcoxon test, Sign test and Rand t-test were evaluated and p values were obtained. CV can be used to assess the class predictability of a model and permutation test can be used to assess the statistical discrimination of a model (Rubingh et al., 2006). Permutation test provides an objective assessment of the stability and performance of the model when the dimensionality (number of samples and data) is appropriate (Rubingh et al., 2006).

## Data Availability

All data produced in this study are included in the published article and its supplementary information, or are available from the lead contact upon request. This paper does not report original code. Any additional information required to reanalyze the data reported in this paper is available from the lead contact upon request.
